# Supplementary material for: Content-rich biological network constructed by mining PubMed abstracts
Source: BMC Bioinformatics. 2004 Oct 8;5:147. doi: 10.1186/1471-2105-5-147 (PMC528731; doi:10.1186/1471-2105-5-147)
Supplement: Additional File 2 — The original results of the above study (non-essential files are deleted to keep the file size under the limit set by BMC bioinformatics). [file 1471-2105-5-147-S2.bz2 › chilibotAdditionalFile2/dip05/649ID10368269E11037/html/HYDA_HYDB.html]

 


 **HYDA** and **HYDB** 
  
Found 12 abstracts in PubMed, retrieved 05.  
 

 What does Google say? 
 PDF only 
| .edu only 

---

**Interactive relationship** (e.g. stimulation, inhibition, etc)

**Non-interactive relationship** (e.g. studied together, co-existance, homology, etc.)

- The results suggest that  **HydA**  and  **HydB**  are translocated across the membrane by the Tat twin arginine translocation system.  Ref: 10525739 Arch Microbiol< MedlineTA>Arch Microbiol,
- The genes encoding the subunits were clustered in a single operon in the order hydC gamma,  **hydB**  beta, and  **hydA**  alpha.  Ref: 10482784 Biochim Biophys Acta, 1999
- **hydA**  and  **hydB** , the genes encoding the large 46 kDa and small 13.  Ref: 10217791 J Bacteriol, 1999
- The translocation of  **HydA**  and  **HydB**  as well as the maturation of the precursor protein of  **HydA**  appear to depend on the presence of the twin arginine motif.  Ref: 10525739 Arch Microbiol< MedlineTA>Arch Microbiol, 1999
- This is encoded by three genes, hydC gamma,  **hydB**  beta and  **hydA**  alpha, organized within a single operon that contains five additional open reading frames ORFs.  Ref: 12605255 J Biol Inorg Chem, 2003
- Mature  **HydB**  and the precursor protein of  **HydA**  were located exclusively in the cytoplasmic cell fraction of the mutant, which catalyzed the reduction of benzyl viologen by H 2, suggesting that  **HydB**  contained Ni.  Ref: 10525739 Arch Microbiol< MedlineTA>Arch Microbiol, 2003
